# GBDKVA score: a scoring system for preoperative risk assessment of adrenal tumors ≤6cm

**DOI:** 10.3389/fendo.2025.1418535

**Published:** 2025-03-17

**Authors:** Siwei Yang, Xianrui Yang, Zhili Yao, Qi Guo, Zekai Hou, Chenyu Wang, Ronghao Cui, Zhun Wang, Gang Li, Changyi Quan, Yuanjie Niu, Yan Zhao, Shaosan Kang, Qiliang Cai

**Affiliations:** ^1^ Department of Urology, Tianjin Institute of Urology, Second Hospital of Tianjin Medical University, Tianjin, China; ^2^ Department of Radiology, Second Hospital of Tianjin Medical University, Tianjin, China; ^3^ Department of Urology, North China University of Science and Technology Affiliated Hospital, Hebei, China; ^4^ Department of Urology, Tianjin Medical University General Hospital, Tianjin, China; ^5^ Department of Endocrinology, Second Hospital of Tianjin Medical University, Tianjin, China

**Keywords:** adrenal tumor, laparoscopic surgery, surgical risks, scoring system, surgical safety

## Abstract

**Objectives:**

To propose a comprehensive scoring system for adrenal tumors ≤ 6cm and evaluate its rationality and validity.

**Materials and methods:**

This study retrospectively analyzed 268 patients with primary adrenal tumors who underwent laparoscopic surgery from January 2018 to December 2022 and all of them met the inclusion criteria. Evaluation scores were calculated for gender (G), body mass index (BMI) (B), tumor diameter (D), the relative position of the tumor to the kidney (K), the relative position of the tumor and the blood vessels (V) and the tumor location in the adrenal gland (A). Then, the total scores were correlated with the operation results, so as to verify the feasibility of GBDKVA in evaluating the surgical risk.

**Results:**

The GBDKVA score showed a consistent and statistically significant correlation with operation time (OT) and blood loss (BL), as well as a correlation with postoperative complications in patients (p < 0.01), but no significant correlation was found with recovery time of gastrointestinal function, bed rest days, indwelling drainage tube days and postoperative hospitalization time were opposite.

**Conclusions:**

GBDKVA score is reliable for preoperative risk assessment of patients with adrenal tumors ≤6cm.

## Introduction

Adrenal incidentalomas (AI) refers to adrenal masses with a diameter larger than 1cm that are unpremeditatedly discovered during abdominal computed tomography (CT) or magnetic resonance imaging (MRI) scans due to other reasons (1). In clinical practice, it was found that the incidence of adrenal masses was about 2% (ranging from 1% to 8.7%), which could originate from the adrenal cortex, medulla or extra adrenal metastasis (2). AI has a very low prevalence of malignancy, less than 3% (3), for example, adrenal cortical carcinoma is an extremely rare endocrine malignancy with poor prognosis (0.3% of adrenal tumors) (4, 5). Nevertheless most AI found in clinic are benign tumors, among which adrenal cortical tumor (a functional adrenal tumor) is the most common (81%-88%), and pheochromocytoma is rare that accounts for 4%-5% of incidentalomas (6–9).

Surgery is the preferred treatment method for patients with functional or malignant tumors larger than 4cm in imaging examination (1, 6). Minimally invasive surgeries such as laparoscopic adrenalectomy have been widely applied in clinical practice for their higher feasibility and safety. And laparoscopic adrenalectomy has become the main treatment method for adrenal masses due to its advantages of reducing hospital stay, recovery time, and postoperative complications (10–12). Previous studies have shown that the body mass index (BMI) of patients is a risk factor for adrenal surgery that is positively correlated with OT and the incidence of postoperative complications (13, 14). And the diameter of the tumor can also affect the occurrence of postoperative complications (15, 16). In addition to the above factors, we also found that the gender of patients, the location of the tumor and the relationship between the tumor and adjacent great blood vessels may also affect the progress of the operation, thereby increasing the risk of surgery.

Therefore, the purpose of this study is to comprehensively analyze the above risk factors and put forward a quantitative scoring system for primary adrenal tumors with a diameter of ≤ 6cm, so as to evaluate the difficulty before operation, predict the blooding risks encountered during operation and make corresponding preparations.

## Materials and methods

### Patients

There were 665 patients with adrenal gland pathological results from January 2018 to December 2022 followed the Helsinki Declaration. The exclusion criteria were shown in **Supplementary Figure 1**. After excluding patients with incomplete data (276), non-single adrenal surgery (71), metastatic malignant tumor (11), Da Vinci robotic laparoscopic surgery (2) and tumors larger than 6cm (37), 268 patients with primary adrenal tumors were evaluated.

Retroperitoneal laparoscopic adrenalectomy was performed in all cases. Before surgery, CT or enhanced CT examination was performed to confirm the existence of adrenal tumor and its related anatomical features. All patients underwent related laboratory tests including five items of hypertension (renin, angiotensin II, aldosterone, adrenocorticotropic hormone and cortisol) and blood electrolytes. Postoperative follow-up of the patients was carried out according to the standards of Guidelines for the Diagnosis and Treatment of Urology and Andrology Diseases in China.

### GBDKVA score

**Figure 1:**
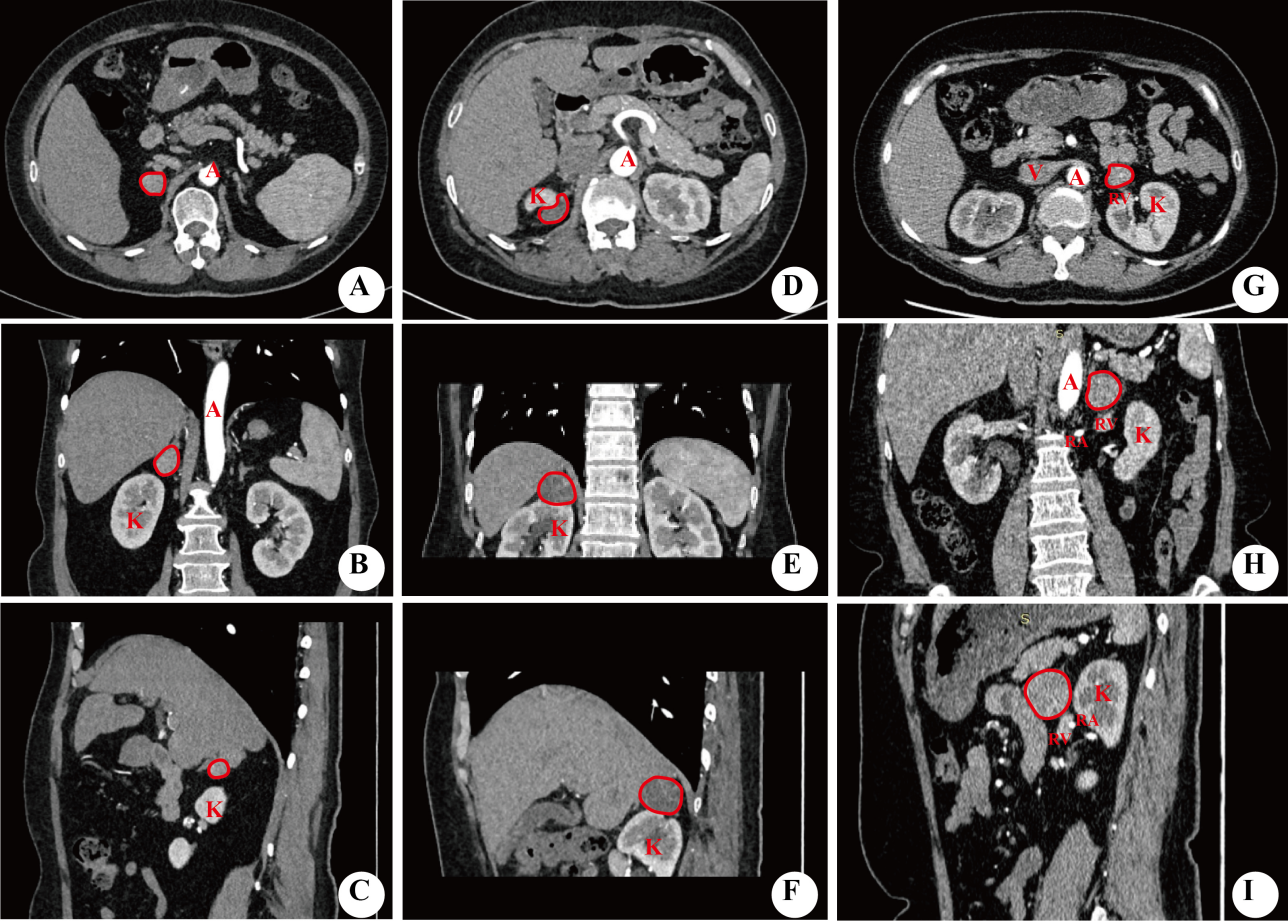
Imaging of relative position of the tumor to the kidney. **(A–C)** showed the tumor located above the upper pole of kidney. **(D–F)** showed the tumor located at the upper pole of the kidney, and **(G–I)** showed the tumor located in the renal hilum. All figures were imaged in transverse, coronal, and sagittal planes. A, abdominal aorta; K, kidney; RA, renal artery; RV, renal vein; V, inferior vena cava.

**Figure 2:**
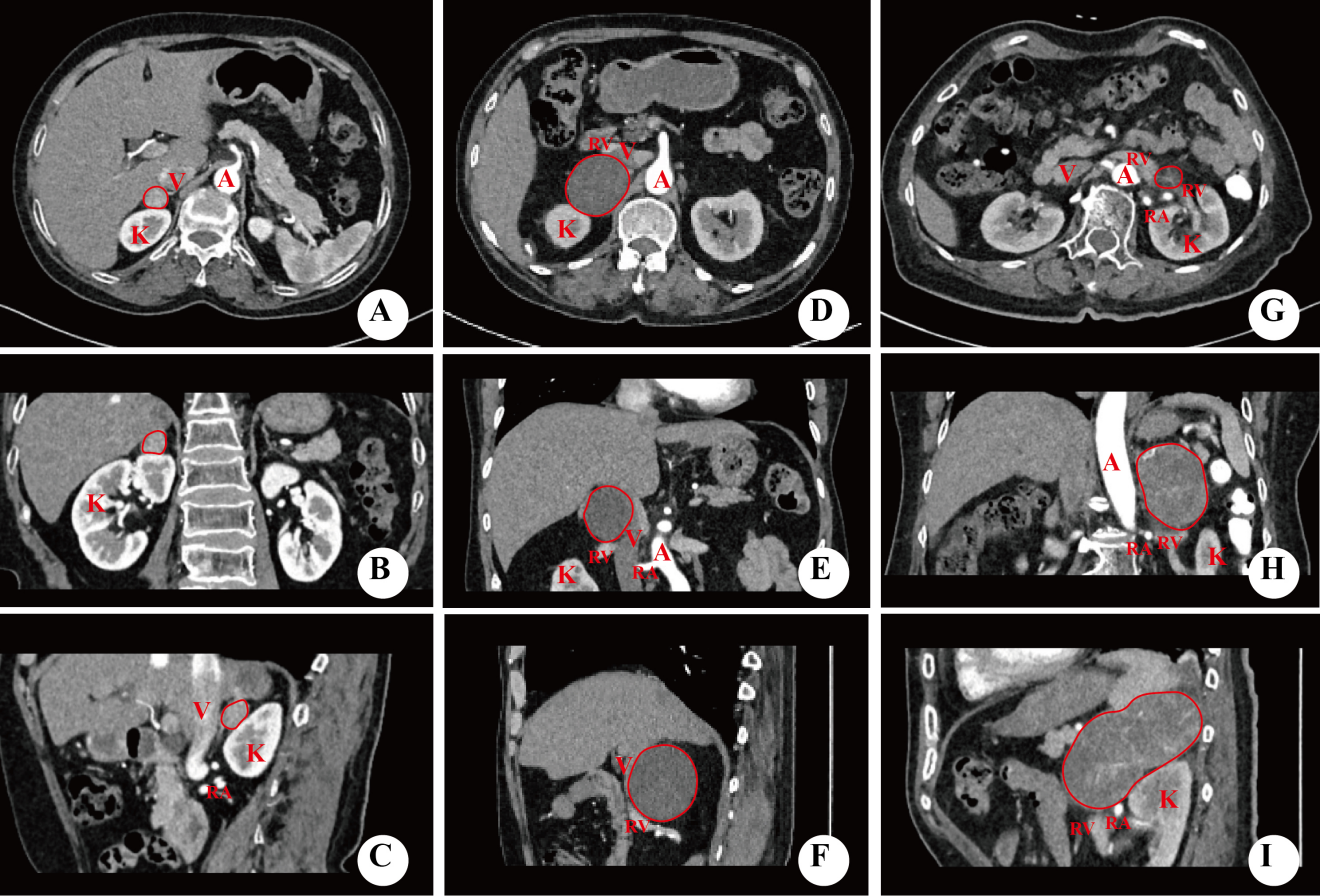
Imaging of the relative position of tumor and blood vessel. **(A–I)** were schematic diagrams of the tumor without squeezing blood vessels, squeezing blood vessels and enveloping blood vessels respectively. All figures were imaged in transverse, coronal and sagittal positions. A, abdominal aorta; K, kidney; RA, renal artery; RV, renal vein; V, inferior vena cava.

**Figure 3:**
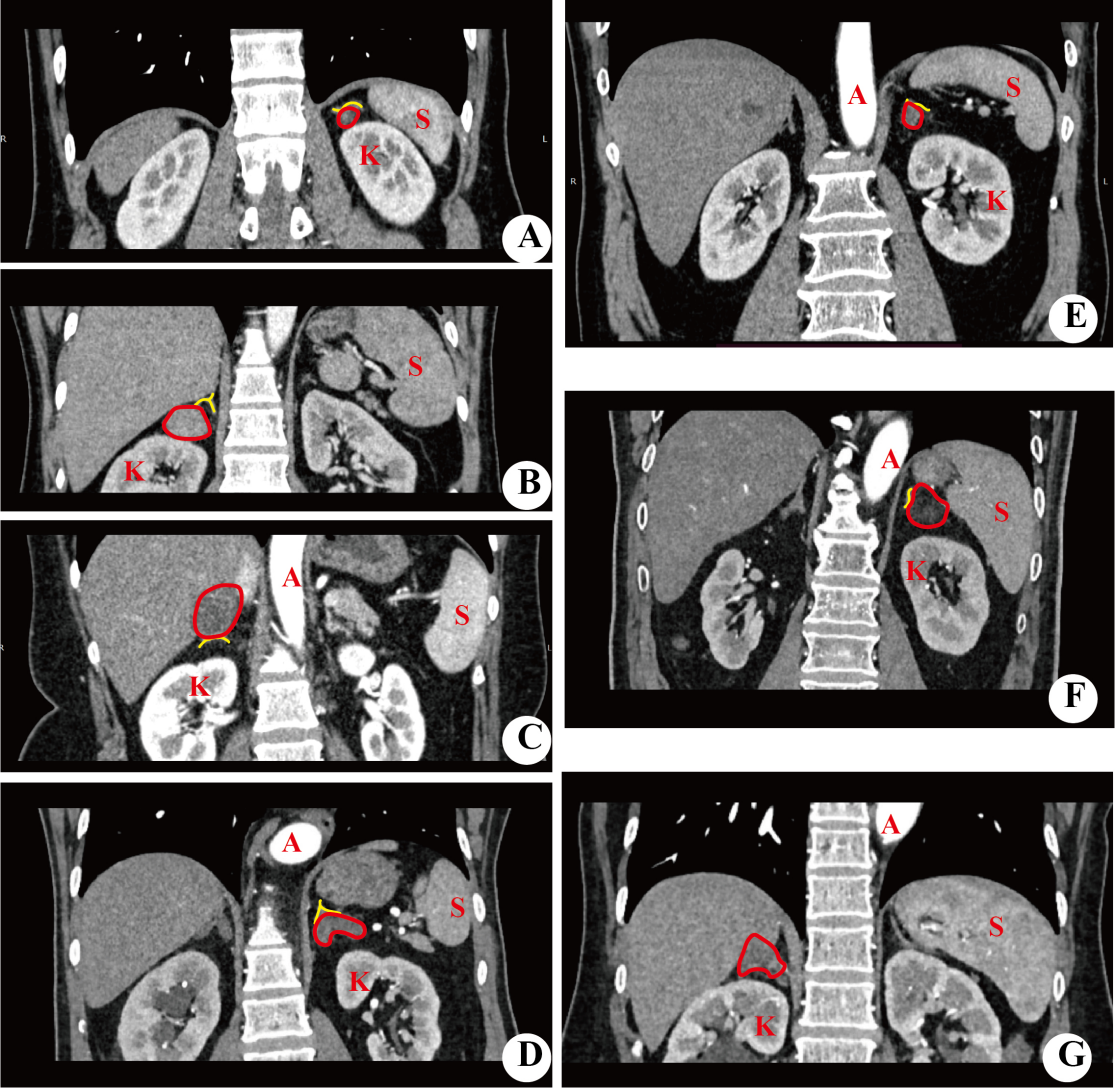
Imaging of the tumor location in adrenal gland. **(A–D)** showed that the tumor originated from medial ramus, lateral ramus, bifurcation, medial and lateral ramus. **(E–G)** showed the tumor was located in unilateral ramus and bifurcation, bilateral ramus and bifurcation. All figures were coronal imaging. A, abdominal aorta; K, kidney; S, spleen.

We define the combination of letter abbreviations for the six sections of this score as the name of the scoring system, that is called the GBDKVA scoring system. Its definition is as follows: G represents the gender of patients; B represents BMI; D is tumor diameter; K represents the relative position of tumor and anatomical structures of kidneys; V is the relative position of tumor and blood vessel; A represents the tumor location in the adrenal gland. The items of G and A are divided into 1,2 points, while B, D, K and V are divided into 1,2,3 points. The weighting of this scoring system relies mainly on the discussion and consensus of experts in the relevant fields, including urologists, endocrinologists, and imaging physicians, who assess each scoring element based on their extensive clinical experience. The specific scoring criteria are shown in **Table 1**, **Figures 1**–**3**. According to this scoring system, the median scores of women and men are 8.00 [7.00, 9.00] and 9.00 [8.00, 9.00]. And the total score is 9.00 [8.00, 10.00].

**Table 1:** Composition and standard of GBDKVA scoring system.

GBDKVA Scoring System for Adrenal Tumors ≤6cm			
GBDKVA Score	1	2	3
**G**. Gender	Female	Male	—
**B**. BMI ,kg/m^2^	<25	≥25, <30	≥30
**D**. Diameter, cm	≤2	>2, ≤4	>4, ≤6
**K**. Relative position of the tumor to the kidney	Above the upper pole of kidney	At the upper pole of kidney	Renal hilum
**V**. Relative position of tumor and blood vessel	None	Tumor squeezes blood vessels	Tumor envelopes blood vessels
**A**. The tumor location in the adrenal gland^*^	Medial ramus. Lateral ramus. Bifurcation. Medial and lateral ramus.	Unilateral ramus and bifurcation. Bilateral ramus and bifurcation.	—

*: The four types of relative position of tumor and adrenal structure were all 1 point: the tumor was located in the medial ramus, or lateral ramus, or bifurcation, or medial and lateral ramus. The tumor located in the adrenal collateral ramus (whether unilateral or bilateral) and bifurcation was scored as 2 points.

### Data collection

Demographic information of the patient was collected from the past medical records by three urological surgeons. Prior to imaging data collection, two data collectors were given standardized training by a specialist imaging doctor and were asked to score the data according to a uniform scoring rubric. The relative position of the tumor was entered according to adrenal CT plain scan or enhanced CT. Clinical baseline data, demographic characteristics and GBDKVA score were shown in **Table 2**. Of all the patients, 133 cases (49.63%) had tumors on the left side, including 53 (19.78%) of women and 80 (29.85%) of men. While 68 (25.37%) women and 67 (25.00%) men were on the right. The median maximum tumor diameter of all patients was 3.00 [2.23, 4.00] cm, and that of women and men was 3.00 [2.40, 3.75] and 2.90 [2.20, 4.20]cm respectively.

**Table 2:** Clinical baseline data of this study.

	Female	Male	Total
Demographics			
Quantity, n (%)	121 (45.15)	147 (54.85)	268 (100)
Age, yr	59.00 [47.00, 64.50]	53.00 [41.00, 59.00]	56.00 [44.00, 62.00]
Weight, kg	65.00 [59.50, 71.25]	80.00 [71.00, 89.00]	73.00 [63.25, 83.00]
Height, cm	160.00 [158.50, 165.00]	174.00 [170.00, 177.00]	169.00 [160.25, 175.00]
BMI, kg/m2	24.84 [22.63, 27.34]	26.81 [24.17, 29.07]	26.04 [23.19, 28.41]
Tumor characteristics			
Laterality, n (%)			
Left	53 (19.78)	80 (29.85)	133 (49.63)
Right	68 (25.37)	67 (25.00)	135 (50.37)
Diameter, cm			
Maximum diameter	3.00 [2.40, 3.75]	2.90 [2.20, 4.20]	3.00 [2.23, 4.00]
Tumor pathology, n (%)			
Cortical adenoma	89 (33.209)	95 (35.448)	184 (68.657)
Pheochromocytoma	12 (4.478)	17 (6.343)	29 (10.821)
Adrenal cyst	6 (2.239)	11 (4.104)	17 (6.343)
Myeloid lipoma	3 (1.120)	9 (3.358)	12 (4.478)
Cortical adenocarcinoma	1 (0.373)	1 (0.373)	2 (0.746)
Ganglioneuroma	1 (0.373)	1 (0.373)	2 (0.746)
Schwannoma	2 (0.746)	0 (0)	2 (0.746)
Others	7 (2.612)	13 (4.851)	20 (7.463)
Surgical outcomes			
OT, min	90.00 [70.00, 120.00]	105.00 [75.00, 145.00]	100.00 [75.00, 135.00]
BL, ml	55.00 [30.00, 77.50]	60.00 [45.00, 95.00]	60.00 [40.00, 85.00]
RTGIF, d	2.00 [1.00, 2.00]	2.00 [1.00, 2.00]	2.00 [1.00, 2.00]
BRD, d	2.00 [1.00, 2.00]	2.00 [1.00, 2.00]	2.00 [1.00, 2.00]
IDTD, d	4.00 [3.00, 5.00]	4.00 [3.00, 5.00]	4.00 [3.00, 5.00]
PHT, d	5.00 [4.00, 5.00]	5.00 [4.00, 6.00]	5.00 [4.00, 5.75]
Surgical margin, n (%)			
Negative	121 (45.15)	147 (54.85)	268 (100)
Positive	0	0	0
Complications, n (%)			
Yes	18 (6.72)	33 (12.31)	51 (19.03)
NO	103 (38.43)	114 (42.54)	217 (80.97)
GBDKVA score			
Aggregate score	8.00 [7.00, 9.00]	9.00 [8.00, 9.00]	9.00 [8.00, 10.00]

BMI, body mass index; OT, operation time; BL, Blood loss; RTGIF, Recovery time of gastrointestinal function; BRD, Bed rest days; IDTD, Indwelling drainage tube days; PHT, Postoperative hospitalization time.

Values were expressed in median and interquartile range.

### Statistical analysis

Data was shown as Median and interquartile range (**IQR**). The normality of data distribution in this study was analyzed by Shapiro-Wilk test or Kolmogorov-Smirnov test. Non-parametric rank sum tests called Mann-Whitney U test and Kruskal-Wallis test were used to analyze the statistical difference of operation time (OT) and blood loss (BL) between different scoring groups of GBDKVA. The correlation between each item of the GBDKVA scoring system and OT and BL was tested by Spearman correlation analysis. The Mann-Whitney U test was used to analyze the difference between the two grades of GBDKVA score. The correlation between the total score of GBDKVA and OT or BL was analyzed by linear regression model, and R^2^ greater than 0.5 was considered statistically significant. All the above data were calculated by SPSS (Statistical Package for the Social Sciences 26.0.0. Figures were drawn by GraphPad Prism 8.3.0 and Adobe Illustrator 26.0. The p-value less than 0.05 was defined significance and the tests performed were bilateral.

## Results

### Tumor pathology and surgical results

Pathological examination was conducted in all patients after retroperitoneal adrenalectomy. The results showed that cortical adenoma (184, 68.657%) and pheochromocytoma (29, 10.821%) accounted for about 80% of all cases, and the numbers of male and female were 95 (35.448%), 17 (6.343%) and 89 (33.209%), 12 (4.478%), respectively. Adrenal cyst (17, 6.343%) and myeloid lipoma (12, 4.478%) accounted for 11 (4.104%), 9 (3.358%) and 6 (2.239%), 3 (1.120%) in male and female patients respectively. Both adrenal cortical adenocarcinoma and ganglioneuroma were identified in one male and one female case each (0.373%, 1/268). Two (0.746%) patients with schwannoma were female. The other 20 (7.463%) patients had other pathologies, including hemangioma, teratoma and nodular adrenal hyperplasia. Notably, three cases following unilateral laparoscopic adrenalectomy revealed multiple unilateral adrenal tumors, including cortical adenoma with ganglioneuroma, cortical adenoma with schwannoma, and cortical adenoma with myelolipoma. (**Supplementary Table 1**). And pathological results showed that the surgical margin of the tumor was negative (268,100%).

The OT of a patient was defined as the time from the incision of the skin at the surgical site to the completion of skin suturing. The BL was the blood loss during the operation. All patients were operated under general anesthesia. The median of OT and BL were significantly larger in males (105 vs 90 min; 60 vs 55 mL). The overall OT and BL of patients were 100.00 [75.00, 135.00] min and 60.00 [40.00, 85.00] mL. The median recovery time of gastrointestinal function (RTGIF) and bed rest days (BRD) in all patients (both male and female) after general anesthesia was 2.00 [1.00, 2.00] days. Similarly, the median indwelling drainage tube days (IDTD) for male and female patients and overall patients was identical, which was 4.00 [3.00,5.00] days. And 5.00 [4.00, 6.00], 5.00 [4.00, 5.00] and 5.00 [4.00, 5.75] days were the medians of postoperative hospitalization time (PHT) of male and female patients and the overall patients respectively. In this study, post-operative fever or confirmed infection was defined as perioperative complications of patients. The incidence of complications in men (12.31%) is higher than that in women (6.72%). A total of 51 patients (19.03%) developed postoperative complications, and treated with antibiotics (**Table 2**).

### GBDKVA score results

The researchers divided the six components of the GBDKVA score into groups firstly. We grouped the gender (G) and the tumor location in the adrenal gland (A) into 1 and 2 points. The BMI (B), the maximum diameter (D), relative position of the tumor to the kidney (K) and relative position of the tumor and the blood vessels (V) were divided into 1, 2 and 3 points. The detailed scoring criteria were shown in **Table 1**.

Subsequently, six components of the scoring system were analyzed in the Mann-Whitney U test or Kruskal-Wallis test to compare the difference between OT and BL in different groups. We also analyzed the laterality of the tumor. The data were integrated and analyzed by SPSS 26.0.0, and it was found that there was no significant difference in the distribution of OT and BL between the left and right tumor groups. In the GBDKVA score, there seemed to be no obvious inconsistency in the distribution of OT and BL among different groups of B, D and K, while the grouping of V components was meaningful (OT: p = 0.0005, BL: p = 0.0323). The OT showed a statistically significant gender difference (p = 0.0108), whereas no significant gender difference was observed in BL. For component A, we divided it into two groups. Group 1 indicated that the tumors originated from medial ramus, lateral ramus, bifurcation, medial ramus and lateral ramus were no significant difference in OT and BL proved by Kruskal-Wallis test. The tumor located in unilateral branch and bifurcation, bilateral branch and bifurcation scored 2 points, and no significance was observed. However, it was worth noting that the OT of the two groups of component A was obviously different (p = 0.0223), while no difference was observed in BL. And it was different for the distribution of OT (p = 0.0115) and BL (p = 0.0314) among the scores.

After the above initial grouping, the correlation between GBDKVA score and OT and BL were examined by Spearman correlation analysis. The results indicated that the score of tumor diameter (D) and its true data, as well as the relative position of tumor and blood vessel (V) were consistent with the correlation between OT and BL and had statistical significance (p < 0.05, accurate p-value was shown in **Table 3**). However, neither the score of BMI (B) of patients nor the real data was related to OT and BL. The position of the tumor relative to the kidney (K) also seemed to have irrelevances to OT and BL. The results of Spearman correlation analysis suggested that gender (G) and the tumor location in the adrenal gland (A) were related to OT (p = 0.0106; p = 0.0220), but BL was meaningless. Particularly, the correlation of OT and BL was consistent and statistically significant for the total score of GBDKVA system (p < 0.01). We also found that the left and right sides of the tumor were not related to OT and BL (**Table 3**). Then, we analyzed the demographic characteristics of GBDKVA score, and examined the goodness of fit between GBDKVA score and the median of OT and BL (**Supplementary Figure 2**; **Supplementary Table 2**). The results revealed that the numerical value of GBDKVA score fitted well with OT (R^2^ = 0.6923), while R^2^ with BL was less than 0.5 (0.4781).

**Table 3:** Verification of the relationship between tumor laterality, GBDKVA score and OT and BL.

Variable		P value			
		Grouping difference of scoring system^a,b^		Correlation^c^	
		OT	BL	OT	BL
Laterality		NS^a^	NS^a^	NS^a^	NS^a^
GBDKVA score					
**G**	Score	0.0108**^a^**	NS**^a^**	0.0106	NS
**B**	Real data	NA	NA	NS	NS
	Score	NS**^b^**	NS**^b^**	NS	NS
**D**	Real data	NA	NA	0.0189	0.0081
	Score	NS**^b^**	NS**^b^**	0.0389	0.0086
**K**	Socre	NS**^b^**	NS**^b^**	NS	NS
**V**	Score	0.0005**^b^**	0.0323**^b^**	0.0005	0.0320
**A**	Group				
	1	NS**^b^**	NS**^b^**	NA	NA
	2	NS**^a^**	NS**^a^**	NA	NA
	Score	0.0223**^a^**	NS**^a^**	0.0220	NS
Aggregate score		0.0115**^b^**	0.0314**^b^**	0.006685	0.006705
GBDKVA grade		0.0083**^a^**	0.0028**^a^**	0.008101	0.002687

OT, operative time; BL, blood loss; NS, not significant (P > 0.05); NA, not available.

^a^Mann-Whitney U test.

^b^Kruskal-Wallis test.

^c^Spearman correlation analysis.

After the above test, we divided the GBDKVA score into two grades (Grade 1: 6-10 score, Grade 2: 11 or above). Then the Mann-Whitney U test was conducted. No statistical difference was observed in the distribution of OT and BL between the two groups. It was statistically significant to get the correlation between GBDKVA two grades and OT and BL using Spearman correlation test. The p values of the above two test results were less than 0.01, as shown in **Table 3**. Other surgical results such as RTGIF, BRD, IDTD and PHT were compared with GBDKVA score by Spearman correlation analysis. The results indicated no statistically difference. But the score kept statistical significance with the postoperative complications (p = 0.019). The details were shown in **Supplementary Table 3**.

The Adrenalectomy Risk Score (ARS) is an assessment system used to evaluate the risk of mortality in patients with adrenal tumors (17). The correlation between the ARS score and the GBDKVA score was analyzed using Spearman correlation test, and the results showed no statistically significant difference between the two (**Supplementary Table 4**).

## Discussion

Most adrenal surgical diseases are benign, including adrenal cyst, hyperplasia, hemorrhage, necrosis and other non-tumor diseases. The primary goal of surgical treatment, such as excision of pheochromocytoma, is aimed to reduce hormone secretion of endocrine adrenal incidentaloma and the risk of tumor enlargement. However, it is important to note that surgery does not eliminate the risk of tumor recurrence (18). Laparoscopic adrenalectomy has become the standard treatment for most adrenal surgical conditions in recent thirty years (19). In addition to surgical methods, clinicians should also consider many factors in the surgical treatment of tumor patients, including tumor size, whether the tumor has hormonal function, imaging, tumor growth rate and so on. Besides, the study found that the size of AI seemed to be related to its benignancy and malignancy (20–22). Previous studies have found that BMI and tumor size may influence the OT and BL, and tumor size and OT may also be related to postoperative complications (13–16, 23). At the same time, surgeons should be well-versed in the anatomical relationship between adrenal gland and its surrounding tissues (24), so as to minimize potential risks during surgery, reduce the OT and BL and improve the patients’ quality of life. At present, laparoscopic or retroperitoneal adrenalectomy can be considered to treat early or benign adrenal tumors less than 6 cm without infiltration and metastasis, but it is worth noting that the 6 cm is not the upper limit of laparoscopic surgery for primary adrenal tumors (25, 26).

In order to safely and completely remove the tumor and improve the patients’ quality of life, a detailed evaluation system is indispensable. According to the latest 2022 classification of adrenal cortical tumors by the World Health Organization, the Weiss and modified Weiss scoring system, based on postoperative histopathology, can help diagnose benign and malignant adrenal cortical tumors in adults. The nature and prognosis of eosinophilic and mucinous adrenocortical tumors can be assessed by Helsinki scoring system, which relies on postoperative histopathology and immunohistochemical markers. As for the Wieneke system, which is based on histopathology and as a scoring system for evaluating the pediatric adrenocortical tumors (27). And the pheochromocytoma of the adrenal gland scaled score (PASS) is used to distinguish benign from malignant tumors (28).

The above scoring systems could primarily rely on postoperative histopathology and immunohistochemistry to determine tumor biology and prognosis, but there was no assessment for the risk of surgical treatment for patients with adrenal tumor. Therefore, we propose an innovative preoperative evaluation framework for adrenal tumors ≤6 cm called GBDKVA score. The score is uniquely characterized by integrating patient-specific factors (gender, BMI) and evaluating tumor characteristics and its relationship with surrounding structures (diameter, anatomic relationships). These characteristics make it have unique advantages in preoperative risk prediction. This system addresses existing gaps in preoperative applications by quantitatively assessing surgical difficulties, predicting surgical risks, thereby enhancing clinical decision-making for adrenal tumors.

In the scoring process, we quantified each part of the scoring system and then analyzed its correlation with the surgical results of patients to verify whether the GBDKVA scoring system could predict the surgical risk. Our analysis of the GBDKVA scoring system revealed that, contrary to previous reports, patient BMI showed no significant correlation with OT or BL parameters. Similarly, the same was true of the position of the tumor relative to the kidney. This differs from previous studies and may be related to differences in surgical technique or population characteristics. However, there was a good correlation between the tumor and the relative position of blood vessel and adrenal structure. Generally speaking, GBDKVA score is significantly correlated with the OT and BL in patients with adrenal tumors and shows a linear relationship with the median operative time. However, the score has no statistically significant correlation with RTGIF, BRD, IDTD, or PHT. Notably, the comprehensive scoring system is significantly associated with postoperative complications (fever or confirmed infection). This suggests that the GBDKVA score may play a role in predicting postoperative complications. It required a lot of data for further discussion and research. Additionally, when divided into two grade1 and grade 2, both grades showed obvious positive correlation with OT and BL.

Therefore, GBDKVA score has advantages in predicting the surgical results in patients with adrenal tumors, and can more accurately predict OT and BL. Its grading system could provide a more detailed risk assessment, thus helping doctors to make risk assessment before operation and get amply prepared to reduce the risk of surgery. In addition, the correlation between GBDKVA score and ARS score further proves its reliability in evaluating the risk of surgery, which provides support for individualized treatment programs for patients with adrenal tumors, helps doctors optimize preoperative decision-making and improve the safety of surgery. Through this scoring system, surgeons can make personalized treatment plans and improve the prognosis and quality of life of patients.

The limitations of this research are as follows: first, the scoring system was developed based on retroperitoneal laparoscopic adrenalectomy cases, whereas the applicability of the transabdominal laparoscopic approach has not been validated due to differences in anatomical exposure and surgical technique. Second, a Although our sample size is 268, it is still too small to contain all the results. For example, in the third item of the GBDKVA score “Relative position of tumor and blood vessel (V)”, tumor enveloping blood vessels was relatively rare in tumors less than or equal to 6cm, so we take an 8.3 cm tumor image as an illustration in **Figure 3**. This limitation highlights the need to refine the scoring criteria for high-risk anatomical variants through larger, multi-center studies. Moreover, the data of this scoring system only comes from a medical institution and has not been verified externally. Therefore, the following research needs to expand the sample size and further external verification and prospective validation of GBDKVA score. In addition, GBDKVA score is only for tumors ≤ 6 cm in imaging (CT or enhanced CT), and adrenal tumors > 6 cm need further research and verification. With the accumulation of data and continuous refinement, the scoring system will hopefully further optimize risk assessment in adrenal tumor surgery.

## Conclusions

GBDKVA score is a comprehensive scoring system for preoperative risk assessment of adrenal tumors ≤ 6cm, associated with OT, BL and postoperative complications. It is an important step towards individualized preoperative risk assessment in adrenal surgery. The GBDKVA score is a comprehensive scoring system for preoperative risk assessment of adrenal tumors ≤ 6 cm, associated with OT, BL and postoperative complications. Currently, the system requires further improvement, and future studies are needed to consolidate its position in clinical practice by expanding its validation and technical adaptation.

## Data Availability

The original contributions presented in the study are included in the article/**Supplementary Material**. Further inquiries can be directed to the corresponding authors.

## Supplementary material

Supplementary Table 1 The research data of this experiment.

Supplementary Figure 1 Schematic diagram of patient exclusion and inclusion standard.

Supplementary Figure 2 Linear regression analysis of GBDKVA score with OT and BL. OT, operation time; BL, blood loss. R2 was greater than 0.5 was considered statistically significant.
