# Supplementary figures and images for: GBDKVA score: a scoring system for preoperative risk assessment of adrenal tumors ≤6cm
Source: Front Endocrinol (Lausanne). 2025 Mar 17;16:1418535. doi: 10.3389/fendo.2025.1418535 (PMC11955483; doi:10.3389/fendo.2025.1418535)

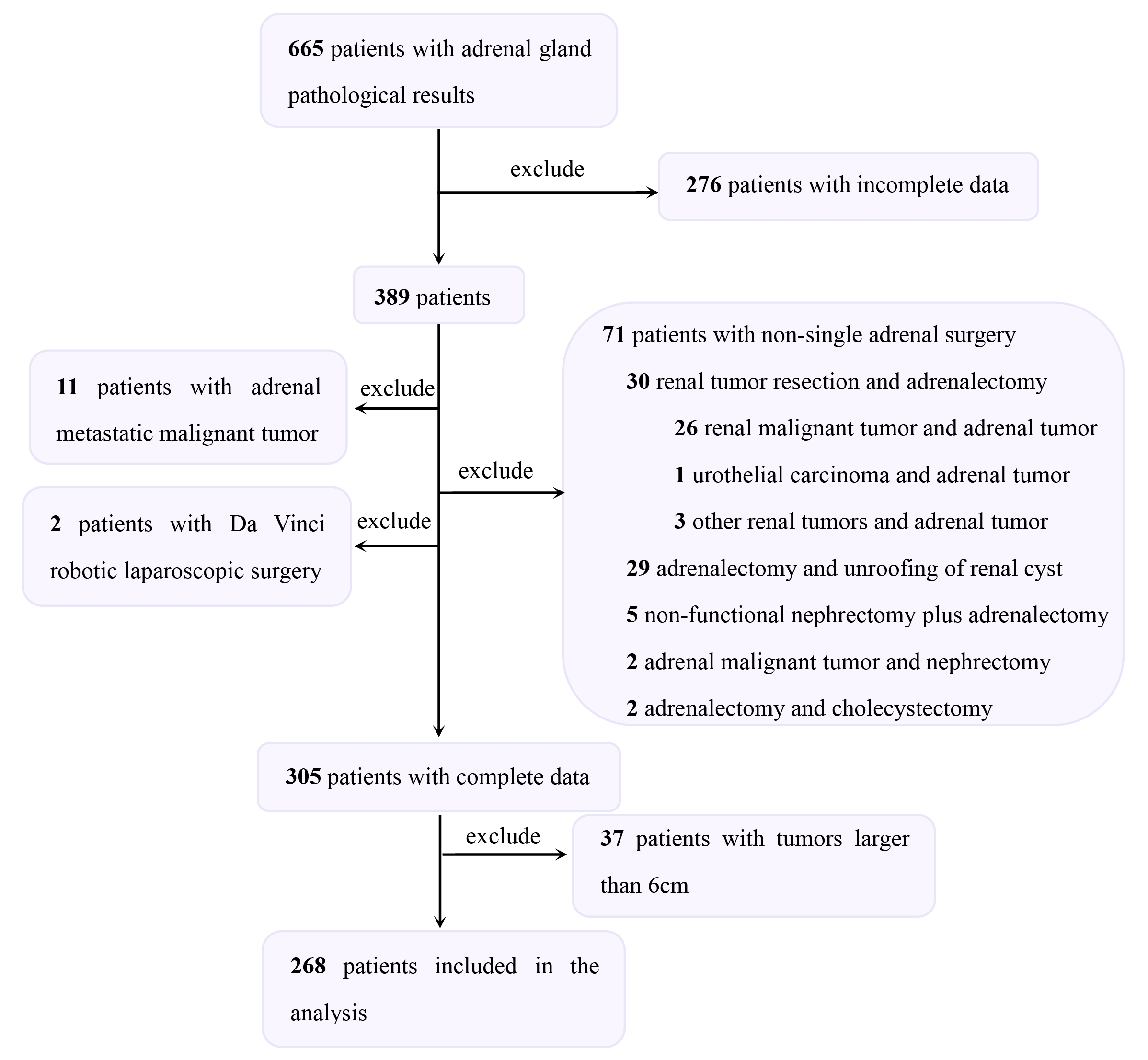

Supplement: Supplementary Figure 1 — Schematic diagram of patient exclusion and inclusion standard. [file Image1.jpg]

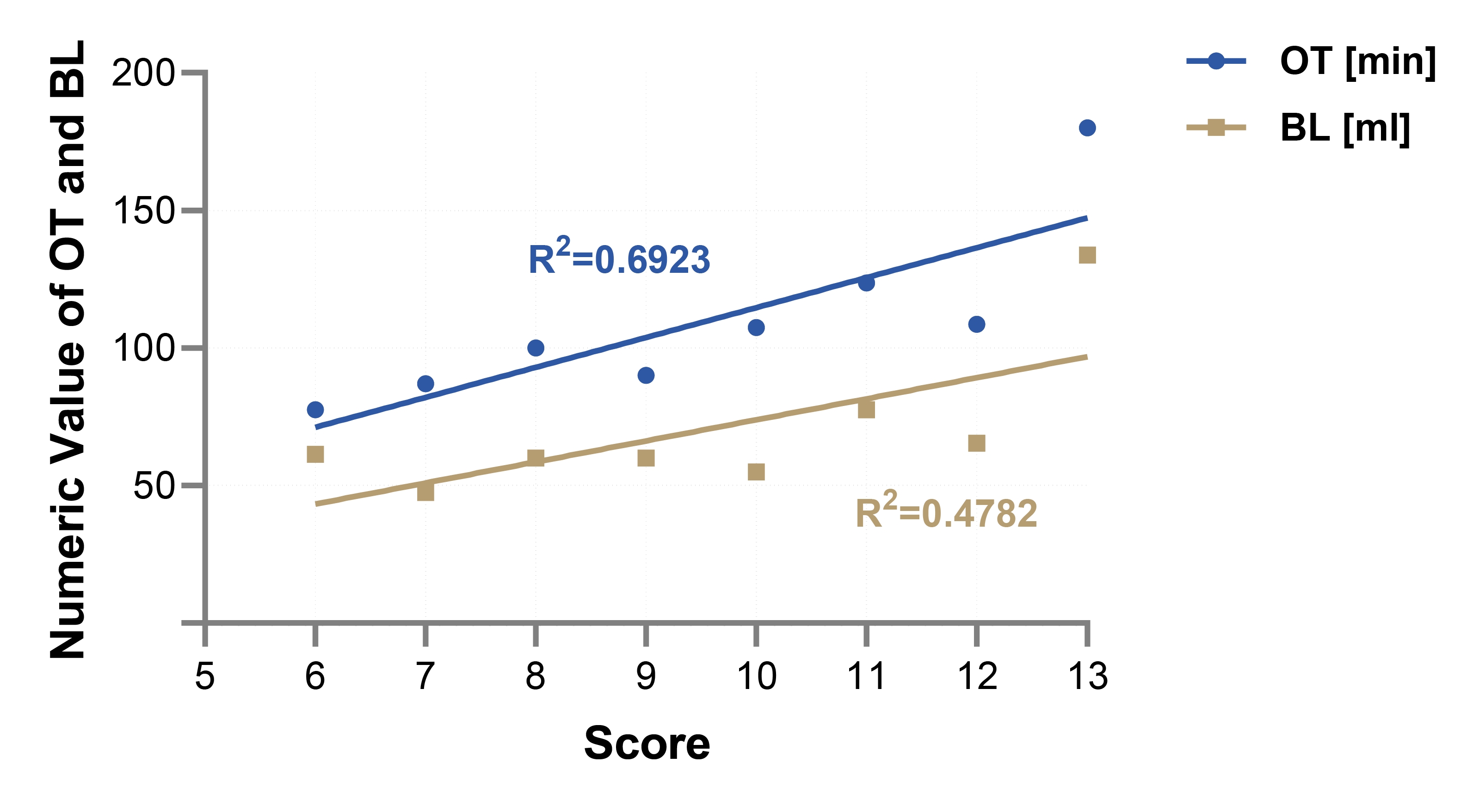

Supplement: Supplementary Figure 2 — Linear regression analysis of GBDKVA score with OT and BL. OT, operation time; BL, blood loss. R2 was greater than 0.5 was considered statistically significant. [file Image2.jpg]
