# Supplementary material for: GBDKVA score: a scoring system for preoperative risk assessment of adrenal tumors ≤6cm
Source: Front Endocrinol (Lausanne). 2025 Mar 17;16:1418535. doi: 10.3389/fendo.2025.1418535 (PMC11955483; doi:10.3389/fendo.2025.1418535)
Supplement: Supplementary file 4 [file Table2.docx]

Supplementary Table 2: Linear regression analysis and demographics of GBDKVA score with OT and BL.

|  | | **Surgical results** | | **Demographic** | | |
| --- | --- | --- | --- | --- | --- | --- |
|  | | **OT, min** | **BL, ml** | **Female, n** | **Male, n** | **Total, n** |
| **GBDKVA score** | 6 | 77.50 [58.75, 111.07 ] | 61.43 [36.25, 72.75] | 6 | 0 | 6 |
|  | 7 | 87.00 [71.25, 110.00 ] | 47.50 [21.25, 68.82] | 27 | 8 | 35 |
|  | 8 | 100.00 [75.00, 130.00] | 60.00 [40.00, 80.00] | 43 | 31 | 74 |
|  | 9 | 90.00 [70.00, 140.00] | 60.00 [35.00, 80.00] | 25 | 49 | 74 |
|  | 10 | 107.35 [75.00, 130.00] | 55.00 [30.00, 97.50] | 14 | 26 | 40 |
|  | 11 | 123.61 [81.75, 150.00] | 77.50 [50.00, 103.75] | 4 | 23 | 27 |
|  | 12 | 108.57 [70.00, 125.00] | 65.35 [53.75, 75.00] | 1 | 6 | 7 |
|  | 13 | 180.00 [165.00, 204.38] | 133.75 [92.50, 175.00] | 0 | 4 | 4 |
| **R²** | | 0.6923 | 0.4781 | - | - | - |

OT: operative time. BL: blood loss. R^2^: R-squared. Values were expressed in median and interquartile range.
