# Supplementary material for: GBDKVA score: a scoring system for preoperative risk assessment of adrenal tumors ≤6cm
Source: Front Endocrinol (Lausanne). 2025 Mar 17;16:1418535. doi: 10.3389/fendo.2025.1418535 (PMC11955483; doi:10.3389/fendo.2025.1418535)
Supplement: Supplementary file 5 [file Table3.docx]

Supplementary Table 3: Verification of the relationship between GBDKVA score and surgical results.

| **Surgical outcomes** | **p-value*** | | | | |
| --- | --- | --- | --- | --- | --- |
|  | RTGIF | BRD | IDTD | PHT | Complications |
| **GBDKVA score** | 0.083 | 0.086 | 0.073 | 0.100 | 0.019 |
|  |  |  |  |  |  |

RTGTF: Recovery time of gastrointestinal function. BRD: Bed rest days. IDTD: Indwelling drainage tube days. PHT: Postoperative hospitalization time. Values were expressed in median and interquartile range.

*: Spearman correlation analysis
