# Supplementary material for: GBDKVA score: a scoring system for preoperative risk assessment of adrenal tumors ≤6cm
Source: Front Endocrinol (Lausanne). 2025 Mar 17;16:1418535. doi: 10.3389/fendo.2025.1418535 (PMC11955483; doi:10.3389/fendo.2025.1418535)
Supplement: Supplementary file 6 [file Table4.docx]

Supplementary Table 4: Correlation analysis between GBDKVA score and ARS score.

|  | **ARS score** |
| --- | --- |
| **GBDKVA score** | p* = 0.005051 |

*: Spearman correlation analysis
